# High Dose AbobotulinumtoxinA for Blepharospasm: A Case Report on the Safety and Efficacy of 500 Units of AbobotulinumtoxinA

**DOI:** 10.5334/tohm.1134

**Published:** 2026-01-19

**Authors:** Kate Santoso, Ava Baghaei, Khashayar Dashtipour

**Affiliations:** 1 Department of Neurology, Loma Linda University Health System, Loma Linda, CA, USA

**Keywords:** Blepharospasm, Botulinum Neurotoxin, Dystonia, AbobotulinumtoxinA

## Abstract

**Background:**

Blepharospasm is a focal dystonia characterized by involuntary eyelid closure that can impair vision and quality of life. Botulinum neurotoxin type A injections are the standard treatment. However, there is substantial variability in dose requirements among patients. Symptom severity ranges from mild, intermittent blinking to forceful, sustained spasms leading to functional blindness.

**Case Report:**

We present two cases of blepharospasm successfully managed with unusually high doses of AbobotulinumtoxinA administered over 8 and 13 treatment cycles, respectively.

**Discussion:**

Both patients achieved sustained symptomatic relief with only mild, self-limiting side effects, underscoring the importance of individualized dosing strategies in clinical practice.

**Highlights:**

- Two patients with refractory blepharospasm were successfully managed with high-dose AbobotulinumtoxinA (500 U every 12 weeks)
- Both patients achieved sustained symptomatic improvement over multiple treatment cycles
- Adverse effects were mild and transient
- High-dose AbobotulinumtoxinA may represent a safe and effective option for patients inadequately controlled with standard doses

## Introduction

Blepharospasm is a focal dystonia characterized by involuntary contractions of the periocular muscles, most commonly the orbicularis oculi [1,2,3]. These contractions cause forced eyelid closure, which can significantly impair vision and daily functioning. Other manifestations include excessive blinking, eye fluttering, and difficulty initiating eyelid opening [4]. Psychiatric comorbidities such as obsessive-compulsive symptoms are frequently observed [5]. In severe cases, blepharospasm can be profoundly disabling, leaving patients functionally blind [4].

First-line therapy involves botulinum neurotoxin (BoNT) injections into the orbicularis oculi and adjacent muscles, typically administered every three months, with the goal of achieving adequate symptom control using the lowest effective dose. Nonetheless, some patients require higher doses for sufficient relief. Premature discontinuation of dose escalation may lead clinicians to mislabel patients as resistant to BoNT when the optimal therapeutic dose has not been reached [6].

While clinical trials have established the safety and efficacy of various BoNT formulations within defined dosing ranges, patient heterogeneity often necessitates individualized strategies [7,8,9,10].

AbobotulinumtoxinA (aboBoNT-A) is not FDA-approved for blepharospasm in the United States; however, several clinical trials in smaller cohorts have demonstrated its efficacy across a broad dosing spectrum, with mean doses of ~200 U, and some patients receiving up to 340 U [7]. Here, we describe two patients with blepharospasm who required unusually high doses of aboBoNT-A (500 U every three months) to achieve sustained benefit without major adverse effects.

## Case Description

### Case 1 presentation

A 77-year-old right-handed woman with a history of anxiety was diagnosed with severe blepharospasm in 2006. Neurological examination was otherwise normal. Symptoms were triggered by eye opening and closing, with breakthrough spasms interfering with driving and watching television. Anxiety exacerbated her blepharospasm but improved after initiation of fluoxetine.

Initial therapy with 50 U of incobotulinumtoxinA (incoBoNT-A) reduced symptoms by ~80% but lasted only one month. At 100 U, breakthrough symptoms occurred during the final 6 to 8 weeks of each interval, impairing mobility and daily functioning. Increasing to 200 U modestly extended benefit to ~8 weeks. Shortening the interval to every six weeks provided limited improvement. Ultimately, treatment was switched to aboBoNT-A, requiring up to 500 U every 12 weeks for consistent control.

**Figure 1:**
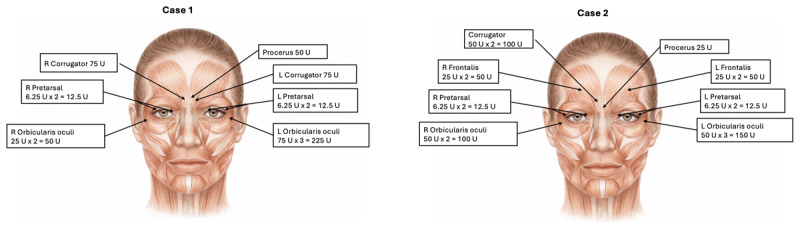
Injection Sites and Doses.

She tolerated the regimen well, reporting only transient, mild blurry vision and diplopia, which resolved within 2–3 weeks. From May 2018 to November 2022, she completed 13 treatment cycles at a dose of 500 U, with sustained benefits. Therapy was briefly interrupted during the COVID-19 pandemic, leading to symptom worsening, but benefits resumed after treatment restart. Injection sites included the procerus, bilateral corrugators, orbicularis oculi, and pretarsal muscles (Figure 1).

### Case 2 presentation

A 63-year-old right-handed woman with hypertension and hyperlipidemia was diagnosed with idiopathic blepharospasm in 2018. She also exhibited mild perioral movements. The neurological exam was otherwise normal.

She had previously received ineffective onabotulinumtoxinA (onaBoNT-A) injections abroad. At our clinic, initial treatment with 137.5 U of aboBoNT-A every two months provided only one month of benefit and mild, self-resolving left ptosis. The dose was increased stepwise (187.5 U → 200 U → 225 U → 350 U → 400 U), with transient blurred vision and mild, short-lived ptosis at higher doses which resolved less than 4 weeks.

Despite escalation, symptom control remained suboptimal until 500 U was administered. At this dose, she achieved consistent symptomatic relief for 12-week intervals. She has now received eight treatment cycles of 500 U, with only minimal, transient side effects and no major adverse events. Injection sites included the procerus, bilateral corrugators, frontalis, orbicularis oculi, and pretarsal muscles (Figure 1).

## Discussion

The American Academy of Neurology recognizes BoNT as the first-line treatment for blepharospasm, supported by both randomized controlled trials and long-term observational data [11]. AboBoNT-A received FDA approval for the treatment of cervical dystonia in 2009 and has since been approved for additional indications, including upper- and lower-limb spasticity [11]. Although it is not FDA-approved for blepharospasm, aboBoNT-A is frequently used off-label for this indication due to its availability and demonstrated efficacy.

Several clinical studies have shown that aboBoNT-A is both safe and effective in patients with blepharospasm, despite relatively small sample sizes. Across these trials, patients experienced statistically significant improvements in symptoms, and treatment was generally well tolerated. The most commonly reported adverse events included ptosis, tearing, blurred or double vision, dry eyes, and facial weakness [12]. Importantly, these studies also demonstrated that effective dosing spans a wide range, from 80 to 340 units.

One double-blind, randomized, placebo-controlled multicenter trial in blepharospasm enrolled 120 patients (85 evaluable) who received a single treatment of aboBoNT-A at one of three doses (40 U, 80 U, or 120 U per eye) or placebo [13]. The primary outcome was functional disability, measured by the Blepharospasm Disability Scale (BDS) using the percentage of normal activity (PNA). By week 4, all three aboBoNT-A dose groups showed significantly greater improvements in functional disability compared with placebo (p ≤ 0.006). Treatment was generally well tolerated, with only mild adverse events reported, including ptosis, blurred vision, and diplopia [13].

A separate randomized, double-blind trial compared aboBoNT-A with onaBoNT-A in 212 patients with essential blepharospasm, using a dose ratio of approximately 4:1 (mean aboBoNT-A dose ~182 U vs. onaBoNT-A ~45 U) [14].The primary endpoint was duration of effect. The mean duration was nearly identical between groups—8.03 ± 4.6 weeks for aboBoNT-A versus 7.98 ± 3.8 weeks for onaBoNT-A—with no statistically significant difference (p = 0.42). Adverse events were more frequent in the aboBoNT-A group (24.1% vs 17.0%) [14].

Another single-blind comparative trial randomized 42 patients with blepharospasm to receive aboBoNT-A or onaBoNT-A [15]. The mean aboBoNT-A dose was ~100 U per session. Primary outcomes included the duration of effect and frequency of booster injections, while secondary outcomes assessed the latency of effect, clinical efficacy (percent improvement on the Blepharospasm Rating Scale), and adverse reactions. Both treatments produced similar efficacy and tolerability. Among patients not requiring booster injections, the duration of effect was slightly longer with aboBoNT-A (13.3 ± 5.9 weeks) compared with onaBoNT-A (11.2 ± 5.8 weeks), though the difference was not statistically significant. Latency of effect, clinical efficacy, and adverse events also showed no significant differences between groups [15].

This variability in dose requirements is not unexpected given the heterogeneity of blepharospasm. Differences in genetic background, severity of spasms, and symptom presentation, ranging from subtle blinking to forceful, disabling contractions, likely contribute to this variability. Comorbidities, concomitant medications, and patient-specific factors, such as perception of benefit and treatment expectations, also play important roles in therapeutic response.

Our two cases highlight the importance of individualized dosing strategies. Both patients required unusually high doses of aboBoNT-A, up to 500 units, to achieve satisfactory symptom control. Dose escalation was approached cautiously, with close monitoring for adverse effects, and both patients tolerated the regimen well. The first patient, a 77-year-old woman with severe blepharospasm and comorbid anxiety, achieved sustained benefit over 13 treatment cycles, with the duration of effect occasionally lasting up to 16 weeks. The second patient, a 63-year-old woman with moderate blepharospasm, demonstrated improvement lasting up to 12 weeks across eight treatment cycles. Neither patient experienced severe adverse effects.

This case series underscores the importance of a patient-centered approach. While prior studies limited aboBoNT-A dosing to 340 units, our findings suggest that certain patients may require and tolerate doses as high as 500 units to achieve optimal outcomes. These observations indicate that carefully monitored high-dose aboBoNT-A may represent a safe and effective therapeutic option for selected patients with refractory blepharospasm.

## Conclusion

This case series demonstrates that high-dose aboBoNT-A (up to 500 U every 12 weeks) can be safe and effective for selected patients with refractory blepharospasm. Although limited to two cases, our findings suggest that individualized, carefully titrated dosing beyond traditional ranges may provide meaningful symptomatic relief in patients inadequately managed with lower doses.

## Data Accessibility Statement

Data will be made available on request.
